# Integrating pre- and post-treatment Plasma Epstein-Barr Virus DNA levels for better prognostic prediction of Nasopharyngeal Carcinoma

**DOI:** 10.7150/jca.56397

**Published:** 2021-03-05

**Authors:** Wanxia Li, Chao Yang, Zehong Lv, Junzheng Li, Zonghua Li, Xiaofei Yuan, Shuting Wu, Yue Yuan, Linchong Cui, Juan Lu, Jing Chen, Feipeng Zhao

**Affiliations:** 1 Department of Otolaryngology-Head and Neck Surgery, Nanfang Hospital, Southern Medical University, Guangzhou 510515, Guangdong, China.; 2 Department of Otolaryngology-Head and Neck Surgery, the Affiliated Hospital of Southwest Medical University, Southwest Medical University, Luzhou 646000, Sichuan, China.; 3 Department of Health Management, Nanfang Hospital, Southern Medical University, Guangzhou 510515, Guangdong, China.; 4 Department of Laboratory Medicine, Nanfang Hospital, Southern Medical University, Guangzhou 510515, Guangdong, China.; 5 School of Traditional Chinese Medicine, Southern Medical University, Guangzhou 510515, Guangdong, China.; 6 Department of Otolaryngology-Head and Neck Surgery, Guangzhou Red Cross Hospital, Jinan University, Guangzhou 510220, Guangdong, China.; 7 Department of Otolaryngology, 942 Hospital of the Chinese People's Liberation Army, Yinchuan750001, Ningxia, China.

**Keywords:** nasopharyngeal carcinoma, plasma, EBV DNA, prognosis, risk stratification

## Abstract

**Background:** Pre- and post-treatment plasma Epstein‐Barr virus (EBV) DNA are important biomarkers for the prognosis of nasopharyngeal carcinoma (NPC). This study was performed to determine the prognostic potential of integrating EBV DNA levels in plasma measured pre-treatment (pre-EBV) and 3 months post-treatment (3 m-EBV).

**Materials and methods:** A total of 543 incident non-metastatic NPC patients treated with intensity-modulated radiotherapy, with or without chemotherapy, were reviewed. Patients were divided into four subgroups based on pre-EBV and 3 m-EBV status. The data for pre-EBV and 3 m-EBV samples were integrated, and the predictability of the survival of patients with NPC was analyzed.

**Results:** There were significant differences in the 5-year progression-free survival, distant metastasis-free survival, locoregional relapse-free survival, and overall survival among the four patient subgroups (*P*<0.001). Patients who tested negative for both pre-EBV and 3 m-EBV had the best prognosis, followed by patients who tested positive for pre-EBV and negative for 3 m-EBV, and those who tested negative for pre-EBV and positive for 3 m-EBV; however, patients who tested positive for both pre-EBV and 3 m-EBV had the poorest chances of survival. Multivariate analyses demonstrated that integration of pre-EBV and 3 m-EBV data was an independent predictor of NPC progression in patients. Receiver operating characteristic curve analysis further confirmed that the combination of pre-EBV and 3 m-EBV had a greater prognostic value than pre-EBV or 3 m-EBV alone.

**Conclusions:** Integrating pre-EBV and 3 m-EBV data could provide more accurate risk stratification and better prognostic prediction in NPC.

## Introduction

Nasopharyngeal carcinoma (NPC) is endemic in Southeast Asia, especially in southern China, and has been established as an Epstein-Barr virus (EBV)-associated cancer 1. Studies have demonstrated that EBV DNA in plasma originates from the tumor, and the load of plasma EBV DNA is strongly correlated with tumor burden 2. In recent years, plasma EBV DNA has been widely used in clinical analysis as a reliable biomarker for screening, monitoring, and prognostic prediction of NPC 3-7. A high pre-treatment plasma EBV DNA load correlates with advanced cancer stages, and poor prognosis 2, 8-11. Conversely, detectable post-treatment EBV DNA levels are indicative of minimal residual disease and have been reported to be a stronger prognostic factor for NPC 2, 5, 12-15.

As pre- or post-treatment plasma EBV DNA is dynamic biomarkers 16, 17, combined evaluation of the changes in both may provide a more accurate prognosis. However, since most of the relevant studies mainly focused on prognosis with either pre- or post-treatment EBV DNA 18-21, to our knowledge, data on the investigation of the prognostic potential of simultaneous temporal changes in pre- and post-treatment plasma EBV DNA level for NPC are limited.

Therefore, we performed this retrospective study to evaluate the prognostic accuracy of the combination of plasma EBV DNA levels measured pre-treatment (pre-EBV) and 3 months post-treatment (3 m-EBV) for risk stratification and prognosis in NPC patients.

## Materials and Methods

### Patients

Data for a total of 543 patients with newly diagnosed, biopsy-proven, and nonmetastatic NPC treated at Nanfang Hospital, of Southern Medical University, from January 2008 to December 2015 were used in this study. Patients whose pre-EBV and/or 3 m-EBV data were not available were excluded. Patients with non-WHO pathological types, distant metastasis at primary diagnosis, and previous or other synchronous malignancies were also excluded. All patients were restaged according to the seventh edition of the American Joint Committee on Cancer (AJCC) staging system based on imaging materials and medical records 22. Our retrospective study was approved by the Ethics Committee of Nanfang Hospital, Southern Medical University (NFEC-2017-165).

### Treatment

All patients were treated with 2.12-2.24 Gy per fraction, with five daily fractions per week, using intensity-modulated radiotherapy (IMRT) for a total of 6-8 weeks. Cumulative radiation doses were 70-74 Gy to the gross tumor target of the nasopharynx (GTVnx), 66-70 Gy to the neck metastatic lymph node area (GTVnd), 60-62 Gy to the high-risk clinical target volume (CTV1), and 50-56 Gy to the low-risk clinical target volume (CTV2). Concurrent chemotherapy (CCT) consisted of cisplatin, administered triweekly, or weekly until the end of radiotherapy. Induction or adjuvant chemotherapy (ICT/ACT) consisted of cisplatin with 5-fluorouracil or cisplatin with taxanes or all three used together, administered triweekly for 2 or 3 cycles. Among the patients, 32 (5.9%) were at stage I and received IMRT treatment alone, 54 (9.9%) were at stage II and received concurrent chemoradiotherapy (CCRT), and 457 (84.2%) were at intermediate and advanced stages III/IV and received CCRT, ICT, and/or ACT.

### Follow-up and Endpoints

All patients were routinely followed up every 3 months within the first year after therapy, every 6 months during the second and third years, and annually thereafter. Physical examination of the head and neck, nasopharyngeal endoscopy, MRI of the nasopharynx and neck, abdominal ultrasound, chest radiography, whole-body PET, and plasma EBV DNA measurements were performed routinely. PET/CT was considered if necessary.

The primary study endpoint was progression-free survival (PFS), which was defined as the time from the initial pathological diagnosis of NPC to relapse at any site or death from any cause, whichever occurred first, or last follow-up visit. The secondary endpoints included distant metastasis-free survival (DMFS, distant metastasis detection, death, or last follow-up visit), locoregional relapse-free survival (LRFS, relapse in nasopharynx or neck lymph nodes, death, or last follow-up visit), and overall survival (OS, all-cause death or last follow-up visit).

### Quantification of plasma EBV DNA

Plasma EBV DNA measurements were performed at pre-EBV and 3 m-EBV stages using a real-time quantitative PCR technique targeting the BamH I-W region of the EBV genome. All plasma EBV DNA assays were conducted at the Laboratory Medicine Center of Nanfang Hospital, Southern Medical University. After the PCR assay, samples with an undetectable EBV DNA signal were recorded as 0 copies/mL, and a positive plasma EBV DNA load was defined as > 0 copies/mL. Referring to previous studies 2, 6, 7, 23-26, the cutoff levels chosen to classify the patients into low and high EBV DNA groups were 1500 copies/mL pre-treatment and 0 copies/mL 3 months post-treatment in this study.

### Statistical analysis

Survival outcomes were estimated using the Kaplan-Meier method and compared by the log-rank test. The Cox proportional hazard model was used for multivariate analysis including the following variables: sex, age (≥45 vs. <45 years), T stage (T_4_ vs. T_1-3_), N stage (N_2-3_ vs. N_0-1_), and the change in pre-EBV and 3 m-EBV. ROC curve analysis was performed to calculate the optimal cut-off value of pre-EBV and 3 m-EBV, and compare the different prognostic values of pre-EBV, 3 m-EBV, and the change in pre-EBV and 3 m-EBV. Statistical analysis was performed using SPSS software version 21.0 (IBM Corporation, Armonk, NY, USA). Two-tailed *P*-values < 0.05 were considered statistically significant.

## Results

### Patient characteristics and survival outcomes

Among the 543 NPC patients, 405 (74.6%) were male and 138 (25.4%) were female, the median age was 45.4 years (range: 13-75). Patient characteristics are listed in **Table 1**. During the median follow-up period of 49.2 months (range: 3-137 months), a total of 177 patients (32.6%) experienced disease progression, including 44 cases of locoregional relapse (8.1%), 95 cases of distant metastasis (17.5%), 24 cases of both locoregional relapse and distant metastasis (4.4%), and 74 deaths (13.6%, 60 patients died from locoregional recurrence or distant metastasis and 14 patients died without locoregional recurrence or distant metastasis). The 5-year PFS, DMFS, LRFS, and OS rates were 66.1%, 75.6%, 86.0%, and 83.8%, respectively.

### Pre- and Post-treatment plasma EBV DNA assessment and survival outcomes

**Figure 1:**
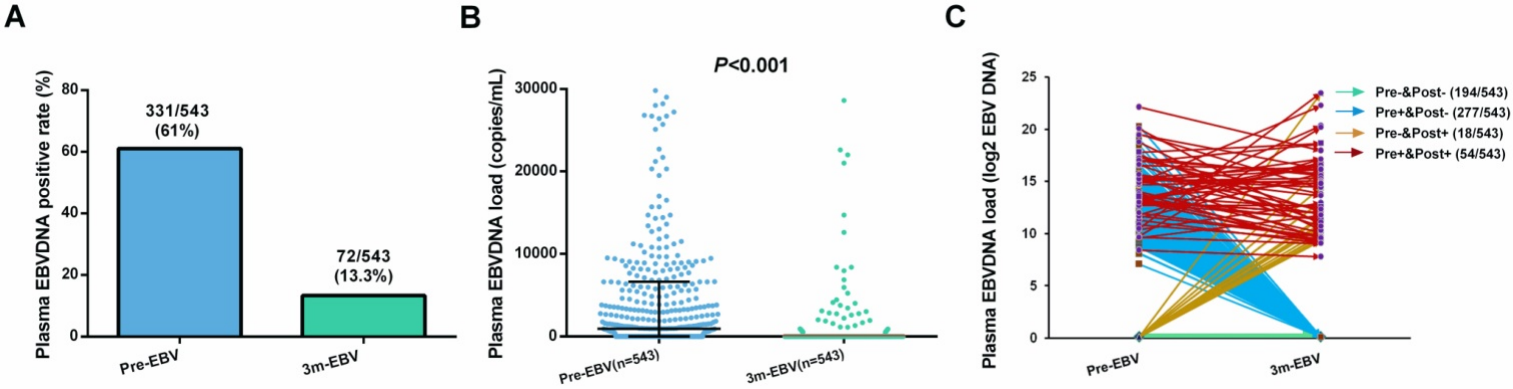
The positive rate and viral load of pre-treatment (Pre-EBV) and 3 months post-treatment (3 m-EBV) plasma EBV DNA. **(A)** Comparisons of the positive rates for Pre-EBV and 3 m-EBV values. **(B)** Comparisons of plasma Pre-EBV and 3 m-EBV values. **(C)** The changes in Pre-EBV and 3 m-EBV DNA levels in plasma.

Of the 543 patients, the positive rate (61.0%) and median viral load (926 copies/mL, range: 0-4.57×10^6^ copies/mL) of pre-EBV samples were significantly higher than those for 3 m-EBV (13.3% and 0 copies/mL, range: 0-1.16×10^7^ copies/mL, respectively) (**Figure 1A and 1B**). Four different patterns were observed in pre-EBV and 3 m-EBV (**Figure 1C**): (1) negative for both pre-EBV and 3 m-EBV; (2) positive for pre-EBV and negative for 3 m-EBV; (3) negative for pre-EBV and positive for 3 m-EBV; (4) positive for both pre-EBV and 3 m-EBV.

**Figure 2:**
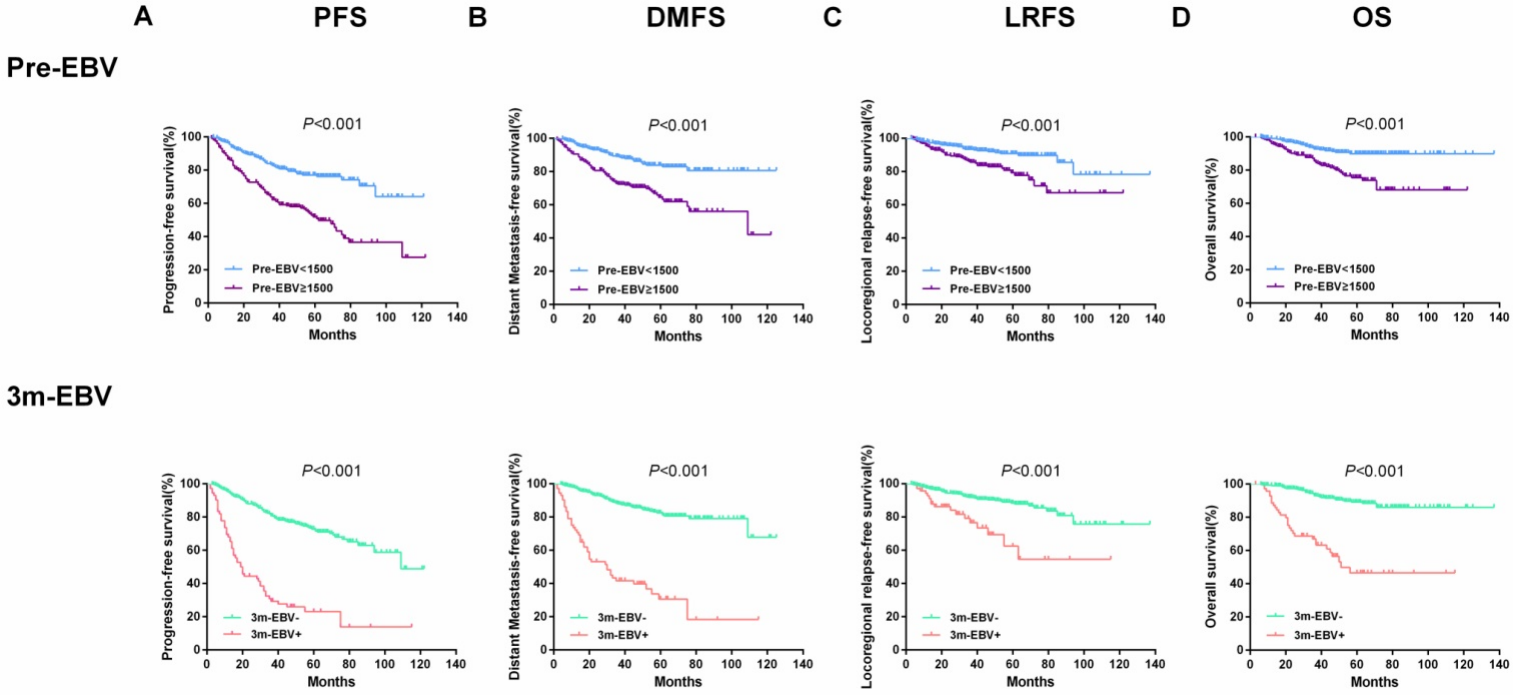
Kaplan-Meier curves of survival outcomes among subgroups defined by pre-treatment (Pre-EBV) and 3 months post-treatment (3 m-EBV) plasma EBV DNA levels. **(A)** Progression-free survival (PFS). **(B)** Distant metastasis-free survival (DMFS). **(C)** Locoregional relapse-free survival (LRFS). **(D)** Overall survival (OS).

Also, the entire cohort of 543 patients was divided into two groups based on plasma EBV DNA cut-off values of 1500 copies/ml for pre-EBV and 0 copies/ml for 3 m-EBV. The results of survival analysis showed that patients with pre-EBV load ≥1500 copies/mL had worse 5-year PFS, DMFS, LRFS, and OS than those with <1500 copies/mL (all *P* < 0.001). Similarly, the 5-year PFS, DMFS, LRFS, and OS were significantly lower among patients with positive (>0 copies/mL) 3 m-EBV than in patients with negative plasma EBV DNA (all *P*<0.001). Kaplan-Meier survival curves for survival analyses of subgroups are shown in **Figure 2**.

### Combination of Pre-EBV and 3 m-EBV data

As the aforementioned analyses showed, both pre-EBV and 3 m-EBV data were effective prognostic factors for NPC patients. Therefore, we stratified the entire population into four subgroups according to the change in the two prognostic factors for pre-EBV and 3 m-EBV: negative for both pre-EBV and 3 m-EBV (Group 1: Pre- and Post-, n=194); positive for pre-EBV and negative for 3 m-EBV (Group 2: Pre+ and Post-, n=277); negative for pre-EBV and positive for 3 m-EBV (Group 3: Pre- and Post+, n=18); positive for both pre-EBV and 3 m-EBV (Group 4: Pre+ and Post+, n=54) (**Table 2**).

**Figure 3:**
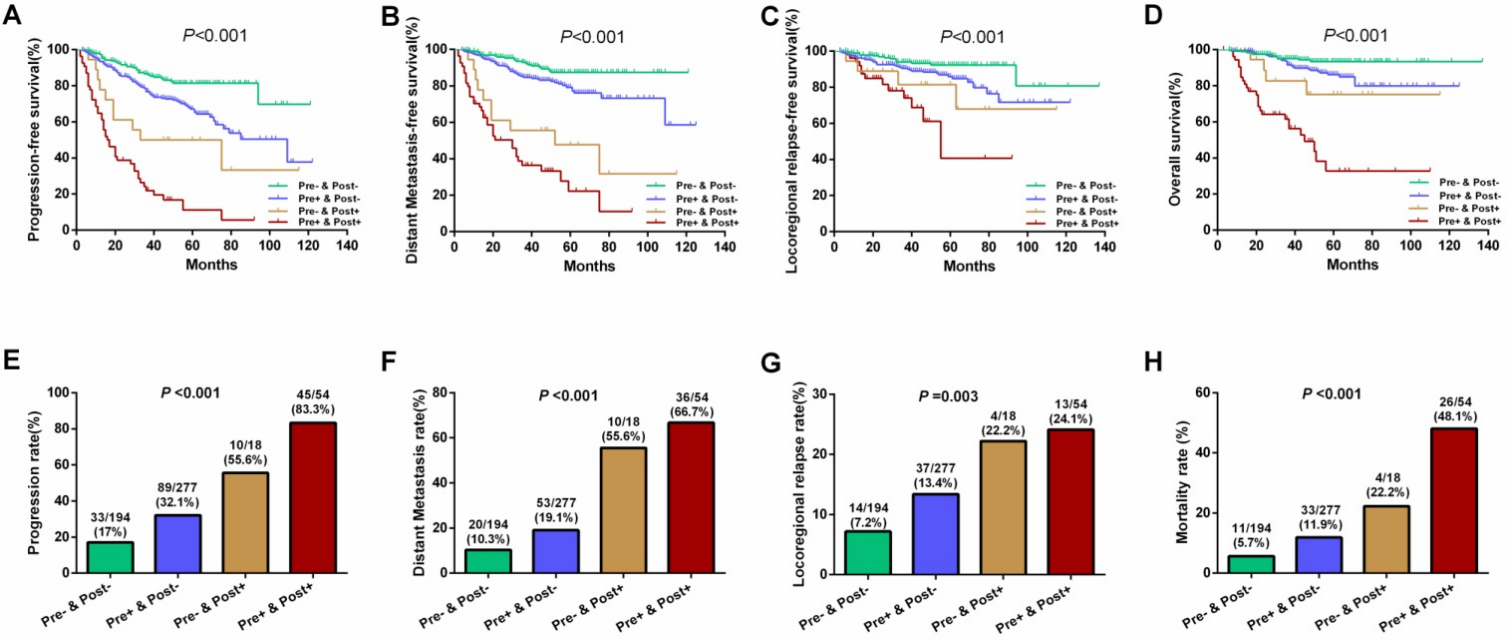
Survival outcomes and treatment failure rates among four patient subgroups according to the combinations of pre- and 3 months post-treatment plasma EBV DNA levels. **(A)** Progression-free survival. **(B)** Distant metastasis-free survival. **(C)** Locoregional relapse-free survival. **(D)** Overall survival. **(E)** Progression rate.** (F)** Distant metastasis rate. **(G)** Locoregional relapse rate. **(H)** Mortality rate. “Pre-” and “Pre+” denote negative and positive pre-treatment EBV DNA status, respectively; “Post-” and “Post+” denote negative and positive 3 months post-treatment EBV DNA status, respectively.

Results of further subgroup prognostic analyses are presented in **Figure 3.** Differences for 5-year PFS (81.4%, 67.1%, 50.0%, and 11.2% for Groups 1 to 4, respectively), DMFS (87.3%, 79.2%, 47.6%, and 22.2% for Groups 1 to 4, respectively), LRFS (92.3%, 85.9%, 81.5%, and 40.7% for Groups 1 to 4, respectively), and OS (93.5%, 86.2%, 75.1%, and 32.8% for Groups 1 to 4, respectively) were statistically significant among the above four subgroups (all *P*<0.001;** Figure 3A-D**). Similarly, the disease progression, distant metastasis, locoregional relapse, and mortality rates were significant among these four subgroups (all* P*<0.05; **Figure 3E-H**). Patients who tested negative for both pre-EBV and 3 m-EBV had the best prognosis, followed by patients who tested positive for pre-EBV and negative for 3 m-EBV, and negative for pre-EBV and positive for 3 m-EBV subgroup patients; patients who tested positive for both pre-EBV and 3 m-EBV had the poorest survival outcomes.

### Cox multivariate analysis

Multivariate analysis revealed that integrating pre-EBV and 3 m-EBV plasma EBV DNA status could be employed as an independent predictor of PFS, DMFS, LRFS, and OS in NPC patients. Compared with the “Pre- and Post-” subgroup, the “Pre- and Post+” and “Pre+ and Post+” subgroups were independent risk factors for worse PFS, DMFS, LRFS, and OS (all *P*<0.001), whereas the “Pre+ and Post-” subgroup was an independent risk factor for comparatively poorer PFS (*P=*0.002) and DMFS (*P=*0.031) (**Table 3**).

Further, the “Pre+ and Post+” subgroup patients had a significantly higher risk of disease progression (hazard ratio (HR), 9.678; 95% confidence interval (CI), 6.019-15.559), distant metastasis (HR, 10.488; 95% CI, 5.924-18.568), locoregional recurrence (HR, 5.628; 95% CI, 2.553-12.404 ), and death (HR, 11.587; 95% CI, 5.576-24.080) than the “Pre- and Post-” group (**Table 3**).

### ROC curve analysis

**Figure 4:**
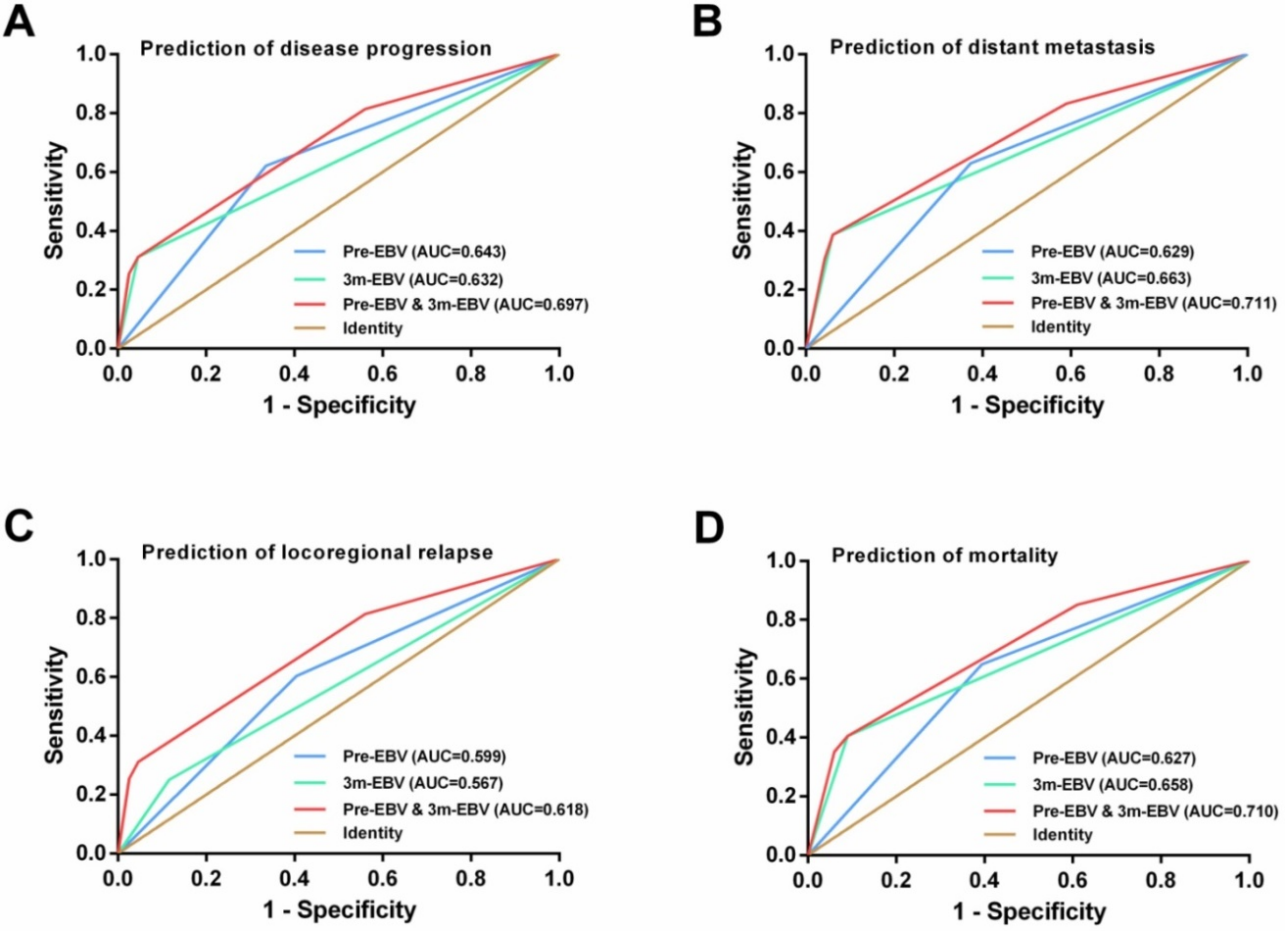
ROC curve analysis for comparing the prognostic potential of pre-treatment (pre-EBV), 3 months post-treatment (3 m-EBV) plasma EBV DNA levels, and integrated pre-EBV and 3 m-EBV values. (A) Prediction of disease progression.** (B)** Prediction of distant metastasis. **(C)** Prediction of locoregional relapse. **(D)** Prediction of mortality. AUC: the area under the curve.

By comparing the ROC curves, integrating the pre-EBV and 3 m-EBV status demonstrated larger area under the curve (AUC) values than pre-EBV or 3 m-EBV alone for predicting NPC progression (AUC=0.697; *P<* 0.001), distant metastasis (AUC=0.711; *P<* 0.001), locoregional relapse (AUC=0.618; *P=*0.002), and mortality (AUC=0.710; *P<* 0.001) (**Figure 4**).

## Discussion

In the past two decades, plasma EBV DNA, an archetypal circulating tumor DNA, has been recognized as a robust biomarker for NPC 27. Previous studies have confirmed that pre- and post-treatment plasma EBV DNA have independent prognostic value in patients with NPC 2, 5-15, 28, 29. The above findings have been further confirmed in our research. We also found that there were significant changes in the positive rate and load of plasma EBV DNA after treatment, and these findings led us to investigate whether the integrated pre-EBV and 3 m-EBV data improve prognostic stratification for NPC patients.

To the best of our knowledge, the performance of the combination of pre- and post-treatment plasma EBV DNA for predicting treatment failure in NPC patients has not been fully investigated, partly because of the limited availability of data and the lack of comprehensive subgroup analyses. In this study, we used the long-term follow-up clinical database with a large sample size, and all eligible patients were divided into four subgroups. We found that patients with persistently negative pre-EBV and 3 m-EBV had the best survival outcome, while patients with consistently positive pre-EBV and 3 m-EBV had the worst prognosis. This result is consistent with previous studies 12, 23.

Furthermore, by using the multivariate prognostic model, our data also showed that patients with consistently positive pre-EBV and 3 m-EBV had a significantly higher risk of disease progression, distant metastasis, locoregional recurrence, and death than the persistently negative group (**Table 3**). The most reasonable explanation for the poor prognosis of these patients is that they either had uncontrolled tumors, unfavorable treatment responses, or residual diseases, which may have progressed with high risk 5, 30. Moreover, closer follow-up visits and further intensified therapy or timely salvage treatment might be beneficial for these high-risk subgroup patients, while excessive or non-contributive treatment and examination can be avoided for patients with persistently negative EBV DNA 31, 32.

Another important finding from our study was that the “Pre- and Post+” patients were revealed to have worse 5-year PFS, DMFS, LRFS, and OS than the “Pre- and Post-”, as well as “Pre+ and Post-” patients. Notably, the prognosis of this subgroup of patients remains controversial, mainly because of a limited number of previous studies, carried out with a small sample size of patients 11, 13, 17. Although our results differ from those obtained by Peng et al. 11, they are broadly consistent with the findings obtained by Lin et al. 23 and Li et al. 13. EBV DNA can be detected in NPC tumor cells 33. Also, cell-free EBV DNA can be detected in the plasma of patients with NPC, which may come from necrosis and lysis of tumor cells infected with EBV 34, 35. In our study, 18 patients (8.5%) with positive 3 m-EBV were found among the 212 patients with negative pre-EBV. It may be that EBV infection has occurred in the nasopharyngeal carcinoma tissue of NPC patients in the “Pre- and Post+” group before treatment, but EBV DNA could not be detected in the plasma. Next, we will collect nasopharyngeal tumor tissues and venous blood samples of NPC patients in the “Pre- and Post+” group before and after treatment, and detect EBV DNA in tumor cells and plasma to confirm this possibility.

Additionally, ROC curve analysis further confirmed that integrating pre-EBV and 3 m-EBV had greater prognostic value than pre-EBV or 3 m-EBV analyzed alone, which was similar to the findings obtained by Peng et al. 11. Considering the above results, integrating pre-EBV and 3 m-EBV data yielded a strong independent prognostic factor for NPC patients, highlighting the feasibility and clinical application in NPC prognostic stratification and tumor surveillance post-treatment.

Our study had some limitations as well. First, some potential biases were unavoidable owing to the retrospective design. Second, the population enrolled in the study was considerably small, especially the sample size for the “Pre- and Post+” subgroup. Moreover, all NPC patients originated from one center and there was no validation cohort. Therefore, larger sample-sized, prospective, multi-center, randomized, and controlled clinical studies are required to further validate our findings.

## Conclusions

In summary, the results of the current study demonstrated that integrating pre-EBV and 3 m-EBV data was an effective prognostic predictor for NPC patients, which could further provide more accurate risk stratification. Our study may help guide individual management for NPC patients' in future clinical practice. However, further studies with larger sample sizes and multiple patient origins will be helpful in establishing the reliability of the proposed method.

## Figures and Tables

**Table 1:** Clinical characteristics of NPC patients (n=543)

Characteristic	N (%)
**Sex**	
Female	138 (25.4%)
Male	405 (74.6%)
**Age (years)**	
<45	252 (46.4%)
≥45	291 (53.6%)
**Smoking**	
Yes	322 (49.3%)
No	221 (40.7%)
**WHO pathologic type^1^**	
Keratinizing carcinoma	3 (0.6%)
Differentiated non-keratinizing carcinoma	34 (6.3%)
Undifferentiated non-keratinizing carcinoma	507 (93.1%)
**Overall stage^2^**	
I	32 (5.9%)
II	54 (9.9%)
III	186 (34.3%)
IV	271 (49.9%)
**Tumor stage^2^**	
T_1_	99 (18.2%)
T_2_	92 (16.9%)
T_3_	109 (20.1%)
T_4_	243 (44.8%)
**Node stage^2^**	
N_0_	59 (10.9%)
N_1_	144 (26.5%)
N_2_	295 (54.3%)
N_3_	45 (8.3%)

^1^ Pathologic type according to the 2005 World Health Organization (WHO) classification of tumors. ^2^ According to the 7^th^ edition of the AJCC staging system.

**Table 2:** Subgroups of the change in plasma EBV DNA levels pre-treatment (pre-EBV) and 3 months post-treatment (3 m-EBV)

Timepoints		3 m-EBV	
		Negative	Positive
Pre-EBV	Negative	194 (35.7%)	18 (3.3%)
	Positive	277 (51.0%)	54 (10.0%)

**Table 3:** Multivariate analysis of prognostic factors of NPC patients

Variable	PFS		DMFS		LRFS		OS	
	HR (95% CI)	*P* value	HR (95% CI)	*P* value	HR (95% CI)	*P* value	HR (95% CI)	*P* value
Sex (male vs. female)	1.273 (0.856-1.895)	0.233	1.296 (0.798-2.105)	0.294	0.890 (0.477-1.661)	0.714	1.532 (0.805-2.916)	0.194
Age (≥45 vs. <45)	1.151 (0.852-1.555)	0.360	1.063 (0.736-1.536)	0.744	1.079 (0.664-1.754)	0.759	1.718 (1.054-2.798)	0.030
Smoking (Yes vs. No)	0.996 (0.706-1.404)	0.981	0.983 (0.644-1.500)	0.937	1.171 (0.666-2.061)	0.584	1.000 (0.592-1.687)	0.999
T stage (T_4_ vs. T_1-3_)	1.163 (0.859-1.574)	0.329	1.023 (0.705-1.484)	0.905	1.661 (1.013-2.724)	0.044	1.122 (0.700-1.799)	0.633
N stage (N_2-3_ vs. N_0-1_)	1.256 (0.900-1.753)	0.180	1.646 (1.071-2.531)	0.023	1.024 (0.612-1.716)	0.927	1.702 (0.988-2.931)	0.055
**EBV DNA change subgroup**		<0.001		<0.001		<0.001		<0.001
**Pre- & Post-**	**Reference**	**Reference**	**Reference**	**Reference**	**Reference**	**Reference**	**Reference**	**Reference**
Pre+ & Post-	1.878 (1.252-2.818)	0.002	1.773 (1.053-2.983)	0.031	1.814 (0.970-3.389)	0.062	1.912 (0.959-3.812)	0.066
Pre- & Post+	4.167 (2.032-8.547)	<0.001	7.254 (3.338-15.762)	<0.001	3.388 (1.104-10.400)	0.033	4.582 (1.442-14.554)	0.010
Pre+ & Post+	9.678 (6.019-15.559)	<0.001	10.488 (5.924-18.568)	<0.001	5.628 (2.553-12.404)	<0.001	11.587 (5.576-24.080)	<0.001

Abbreviations: PFS: progression-free survival; DMFS: distant metastasis-free survival; LRFS: locoregional relapse-free survival; OS: overall survival; HR: hazard ratio; CI: confidence interval.

## Abbreviations

3 m-EBV: 3 months post-treatment
AUC: area under the curve
CTV1: clinical target volume
CCRT: concurrent chemoradiotherapy
CCT: Concurrent chemotherapy
CI: confidence interval
DMFS: distant metastasis-free survival
EBV: Epstein‐Barr virus
GTVnx: gross tumor target of the nasopharynx
ICT/ACT: Induction or adjuvant chemotherapy
IMRT: intensity-modulated radiotherapy
LRFS: locoregional relapse-free survival
CTV2: low-risk clinical target volume
NPC: nasopharyngeal carcinoma
GTVnd: gross tumor target of neck metastatic lymph node area
OS: overall survival
pre-EBV: pre-treatment
PFS: progression-free survival
ROC: receiver operator characteristic
